# Advances in Bioactive Dental Adhesives for Caries Prevention: A State-of-the-Art Review

**DOI:** 10.3390/jfb16110418

**Published:** 2025-11-07

**Authors:** Mohammed Zahedul Islam Nizami, Apissada Jindarojanakul, Qiang Ma, Sang J. Lee, Jirun Sun

**Affiliations:** 1 ADA Forsyth Institute, Somerville, MA 02143, USA; 2 Department of Restorative Dentistry and Biomaterials Sciences, Harvard School of Dental Medicine, Boston, MA 02115, USA; 3 Department of Prosthodontics, Faculty of Dentistry, Mahidol University, Bangkok 10400, Thailand; 4 BISCO Inc., Schaumburg, IL 60193, USA

**Keywords:** bioactive dental adhesives, caries prevention, remineralization, antimicrobial adhesives, tooth-adhesive interface

## Abstract

The long-term success of composite restorations largely depends on the performance of dental adhesives at the adhesive–tooth interface. Despite ongoing improvements, secondary caries remains the leading cause of restoration failure, primarily due to the adhesive layer’s susceptibility to hydrolytic degradation, bacterial invasion, and limited biological functionality. This review provides a comprehensive overview of recent advances in bioactive dental adhesives for preventing recurrent caries, focusing on their mechanisms of action, material performance, therapeutic functions, and clinical potential. Bioactive adhesives combine durable bonding with biofunctional benefits, including remineralization, antimicrobial activity, enzymatic inhibition, and support for tissue regeneration. By integrating these properties, they enhance both the durability of the adhesive interface and oral health. Recent strategies include the incorporation of ion-releasing fillers such as calcium phosphate and bioactive glass, antimicrobial monomers such as MDPB and quaternary ammonium methacrylates, enzymatic inhibitors, and hydrolytically stable resin matrices. Together, these components strengthen the adhesive interface and provide biologically active effects to prevent recurrent caries. Although in vitro findings are promising, challenges remain, including limited long-term clinical data, the absence of standardized evaluation protocols, and barriers to clinical translation. Addressing these gaps is essential to ensure predictable clinical outcomes. Bioactive dental adhesives represent a paradigm shift in restorative dentistry, evolving from passive bonding agents to multifunctional therapeutic materials. By combining structural durability with biological protection, they hold significant potential to prevent recurrent caries and improve the long-term success of composite restorations.

## 1. Introduction

Composite restorations are widely used as direct filling materials for dental caries due to their superior esthetics, dentin-like flexural strength, and dependable bonding performance. The adhesive layer forms the primary interface with the dentin substrate, enabling both micromechanical interlocking and chemical bonding [1]. The structural integrity of this layer is crucial for maintaining bond strength, marginal sealing, resistance to hydrolytic degradation, and, ultimately, the long-term clinical success of the restoration [2].

Despite advances in restorative materials, secondary caries remains the leading cause of restoration failure [3,4,5]. Although many commercially available composites incorporate bioactive components such as fluoride release or antimicrobial agents, these strategies provide limited protection for the adhesive interface, the most vulnerable region of the restoration complex [2]. This interface is inherently prone to hydrolytic degradation, mechanical weakness, and lack of biological activity, rendering it susceptible to breakdown, bacterial infiltration, and recurrent caries.

To overcome these challenges, next-generation adhesives are being engineered with enhanced durability and hydrolytic stability through the replacement of degradation-prone monomers such as Bis-GMA and HEMA [6]. Beyond improved mechanical resilience, current strategies emphasize the integration of therapeutic functions into adhesive systems. Bioactive dental adhesives, defined as materials with biological activities beyond their restorative role [7], are now designed to promote remineralization, exert antibacterial effects, and stimulate tissue regeneration [8]. These multifunctional features not only reinforce the adhesive interface but also actively suppress secondary caries, thereby extending the clinical lifespan of restorations.

Recent efforts have centered on developing bioactive adhesive systems incorporating ion-releasing fillers, antimicrobial monomers, enzymatic inhibitors, and hydrolytically stable resin matrices [6,9,10,11]. These strategies aim to improve interface durability through mineral deposition, suppression of enzymatic degradation, and inhibition of bacterial growth. Nevertheless, despite encouraging in vitro outcomes, clinical translation remains constrained by the absence of long-term data, non-standardized testing protocols, and the persistent challenge of balancing bioactivity with mechanical performance [12,13].

Although no extensive reviews have been solely dedicated to this relatively new field, gaps remain in understanding the integration of therapeutic functions with durable adhesive performance. This review addresses these gaps by synthesizing current advances, highlighting material composition, mechanisms of action, clinical relevance, and emerging strategies. By presenting bioactive dental adhesives as multifunctional, smart biomaterials (i.e., adapting and responding to biological or environmental stimuli), this work underscores their potential to transform adhesive dentistry from passive bonding agents into dynamic contributors to oral health.

## 2. Materials and Methods

A comprehensive literature search was conducted to identify original studies, systematic reviews, and scientific reports relevant to bioactive dental adhesives for caries prevention. The search encompassed multiple databases, including PubMed, Scopus, and Web of Science, ensuring coverage of peer-reviewed and high-quality publications. Google Scholar was not used as a primary source to avoid non–peer-reviewed materials. Searches were limited to English-language publications and spanned the period from 1988 through June 2025. Although bioactive adhesives were not commercially available in the early years, this period was included to capture foundational research related to dental adhesives and bioactivity, using keywords such as *dental adhesives*, *bonding agent*, *bioactive*, *bioactivity*, *caries prevention*, *antimicrobial effect*, *remineralization*, and *regeneration*. Boolean operators (AND, OR) were applied to refine and expand the search as appropriate.

Studies were selected if they investigated the properties, mechanisms of action, therapeutic effects, or clinical potential of bioactive materials incorporated into, or relevant to, dental adhesives. Particular emphasis was placed on antibacterial activity, remineralization capacity, enzymatic inhibition, and mechanical durability. Exclusion criteria included non-peer-reviewed publications, conference abstracts, and studies not directly related to bioactive adhesives. The selected literature comprised in vitro and in vivo studies, clinical trials, and review articles. All sources were critically appraised for methodological quality and relevance. Key findings were synthesized thematically and organized according to the review’s main objectives: material composition, functional mechanisms, in vitro and in vivo performance, and translational challenges. This structured approach ensured a rigorous and transparent evaluation of the current landscape and emerging trends in bioactive dental adhesive systems for caries prevention.

## 3. Principle of Bioactive Dental Adhesives

### 3.1. Function of Bioactive Materials

The concept of bioactivity in dental adhesives marks a key advancement in restorative dentistry, extending beyond the primary function of bonding toward active therapeutic engagement with dental tissues. To begin, it is essential to understand the definition of “bioactive”. According to the 2018 consensus on “Bioactive Dental Materials,” restorative materials may be considered bioactive if, in addition to restoring or replacing tooth structure, they actively stimulate or direct specific cellular or tissue responses or regulate interactions with microbiological species [7]. Extending this definition further, Ferracane et al. (2023) identified three main therapeutic functions of bioactive materials: Remineralization, regeneration, and antibacterial activity [8]. **Remineralization** involves the chemical deposition of mineral ions onto the tooth surface [14]. **Regeneration** refers to stimulating the body’s natural ability to repair or regrow damaged tissues [15]. **Antibacterial activity** is defined as the ability to kill bacteria or inhibit their growth and disease-causing potential [15].

While the antibacterial effects of bioactive materials are well-defined and easily distinguishable, the differentiation between remineralization and regeneration is often more nuanced. The following explanation aims to clarify the distinction between these two therapeutic mechanisms. Remineralization is primarily a chemical process in which mineral ions, such as calcium and phosphate, are redeposited onto demineralized inorganic structures like enamel and dentin. This process halts further demineralization, restores mineral content, and creates a protective surface layer, especially on compromised tooth structures [16]. In contrast, regeneration is a biologically driven process that targets the organic components of the tooth, particularly the dental pulp and the dentin–pulp complex. It involves stimulating the body’s natural healing pathways, including the activation of odontoblasts or progenitor cells, to promote the formation of secondary or tertiary dentin [17].

As the term “bioactive” has gained significant attention in recent years, the FDI World Dental Federation released a policy statement in 2021 to further clarify what qualifies a restorative material as bioactive. This statement outlines five essential criteria that restorative materials must meet to be classified as bioactive [18]. These include: (1) a clearly defined mechanism of action, biological, chemical, or both; (2) scientific evidence of bioactivity demonstrated through in vitro or in situ studies; (3) a specified duration of the bioactive effect; (4) no significant adverse biological side effects; and (5) preservation of the material’s primary restorative function.

### 3.2. Mechanisms of Caries Prevention in Bioactive Dental Adhesives

Bioactive dental adhesives offer a multifaceted approach to caries prevention, combining robust mechanical adhesion with biologically active defense mechanisms. These materials interact actively with the oral environment, promoting remineralization, resisting microbial colonization, and stabilizing the adhesive–tooth interface. A key mechanism is the controlled release of therapeutic ions, primarily calcium, phosphate, and fluoride, which supports the remineralization of demineralized enamel and dentin and enhances the formation of fluorapatite, a more acid-resistant mineral than natural hydroxyapatite. Self-etching adhesives modify or remove the smear layer before sealing dentinal tubules, enhancing adhesive penetration, reducing bacterial contamination, and improving bonding efficacy. This sealing also reduces postoperative sensitivity and prevents bacterial ingress into the pulp [19,20,21].

In addition to mineral deposition, bioactive adhesives often incorporate antimicrobial components such as silver or zinc oxide nanoparticles and chlorhexidine [22,23,24]. These agents disrupt bacterial metabolism and biofilm formation at restoration margins, reducing secondary caries. Many formulations exhibit pH-responsive behavior, releasing ions in acidic conditions and buffering local pH to mitigate demineralization [25,26]. Enzymatic inhibitors prevent collagen breakdown by host-derived proteases, preserving the hybrid layer and enhancing long-term bond durability. Together, these mechanisms provide sustained caries protection, particularly for patients with high caries risk [27,28,29].

**Figure 1:**
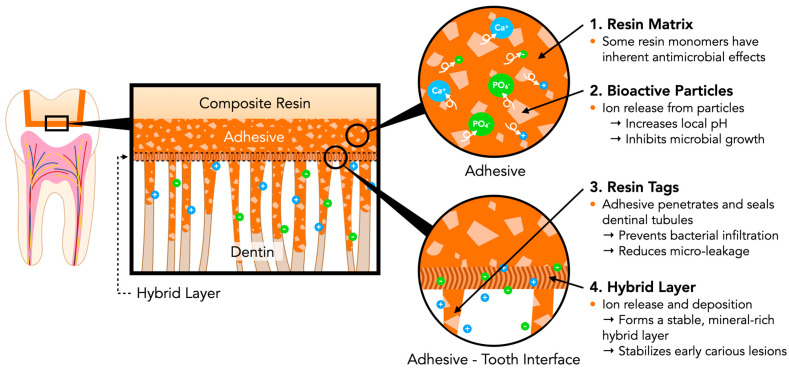
Key Functional Components of Bioactive Adhesives Preventing Recurrent Caries.

The caries prevention mechanisms include ion release, pH buffering, antimicrobial action, and stabilization of the hybrid layer at the adhesive–tooth interface, as discussed in Section 3.2.1, Section 3.2.2, Section 3.2.3 and Section 3.2.4. Collectively, these properties promote tooth repair and inhibit bacterial growth. Figure 1 illustrates the key functional components of bioactive adhesives preventing recurrent caries.

#### 3.2.1. Bioactive Ion Release and Mineral Deposition at the Adhesive–Tooth Interface

One of the primary mechanisms by which bioactive dental adhesives prevent caries is the sustained release of therapeutic ions, namely calcium (Ca^2+^), phosphate (PO_4_^3−^), and fluoride (F^−^), at the adhesive–tooth interface. These ions facilitate the nucleation and growth of hydroxyapatite or fluorapatite crystals, which are more acid-resistant than natural tooth minerals, promoting remineralization of demineralized enamel and dentin. Materials such as bioactive glass (e.g., 45S5 Bioglass) and nano-hydroxyapatite (nHAp) are commonly used to enable ion release, particularly under acidic, caries-prone conditions [30,31,32]. This mineral deposition reinforces the structural integrity of the adhesive–tooth interface, forms a stable, mineral-rich layer, and seals dentinal tubules, reducing postoperative sensitivity. It also fills micro-gaps, minimizing bacterial infiltration and microleakage.

The pH-responsive nature of many adhesives ensures that ion release occurs during cariogenic attacks, providing targeted remineralization precisely when needed [20,33]. Over time, this continuous ion exchange creates a dynamic, self-repairing environment that enhances the long-term durability of restorations. By stabilizing early carious lesions and reducing demineralization without invasive intervention, these adhesives align with the principles of preventive and minimally invasive dentistry. Overall, ion release and mineral deposition protect the restored tooth, promote healing and regeneration, and position bioactive dental adhesives as multifunctional agents in restorative care.

#### 3.2.2. Dynamic pH-Responsive Buffering Function

A critical caries prevention mechanism of bioactive dental adhesives is their pH-responsive buffering capacity, which allows them to counteract the acidic conditions produced by cariogenic bacteria. When oral pH drops below the critical threshold (around pH 5.5), enamel and dentin become prone to demineralization. Advanced adhesives incorporate pH-sensitive components such as bioactive glass, calcium silicate, or phosphate-based fillers, which detect acidic shifts and release alkaline ions like calcium (Ca^2+^), phosphate (PO_4_^3−^), fluoride (F^−^), or strontium (Sr^2+^) in response [25,34,35,36]. This controlled ion release neutralizes acidity at the adhesive–tooth interface, creating an environment less favorable for demineralization and bacterial growth. The buffering effect also helps restore a neutral or slightly basic environment, promoting remineralization.

Unlike conventional adhesives, these smart, self-regulated systems activate only during acid challenges, preserving their therapeutic content and extending functional life. By modulating pH in real time, pH-responsive adhesives maintain hybrid layer stability and reduce marginal degradation and secondary caries formation. This stimulus-responsive behavior exemplifies the transition from passive bonding agents to interactive materials that actively support tooth preservation [37].

#### 3.2.3. Inhibition of Microbial Adhesion and Biofilm Formation

Another key mechanism by which bioactive dental adhesives prevent caries is the inhibition of bacterial adhesion and biofilm formation at the adhesive–tooth interface. Acidogenic bacteria, such as *Streptococcus mutans*, adhere to tooth surfaces and form biofilms that produce acids, leading to demineralization of enamel and dentin. To counteract this, bioactive adhesives incorporate antimicrobial agents as additive particles or functional monomers within the resin matrix, including silver nanoparticles (AgNPs), quaternary ammonium compounds (QACs), zinc oxide (ZnO), chlorhexidine, and chitosan. These agents disrupt bacterial membranes, inhibit metabolic activity, and prevent initial bacterial adhesion [22,38,39,40,41,42,43]. In addition to direct antimicrobial effects, many agents demonstrate contact-killing or ion-mediated actions [44]. For example, silver and zinc ions released over time inhibit bacterial growth without traditional antibiotics, providing sustained long-term antimicrobial protection, particularly at restoration margins where secondary caries often develops [27,45,46]. By reducing bacterial colonization and biofilm formation, bioactive adhesives preserve the adhesive interface and surrounding tooth structure, maintain cleaner restoration surfaces, lower caries risk at margins, and extend the longevity of restorations.

#### 3.2.4. Stabilization of Hybrid Layer to Enhance Bonding Durability

The long-term efficacy of bioactive dental adhesives in caries prevention depends on the stability of the hybrid layer, which is crucial for durable bonding. The hybrid layer forms when adhesives infiltrate demineralized dentin, creating a micromechanical bond between tooth structure and restorative material. However, it is susceptible to enzymatic degradation by host-derived enzymes such as matrix metalloproteinases (MMPs) and cathepsins. Without adequate protection, this degradation can weaken the adhesive bond, create micro-gaps, and allow bacterial infiltration, potentially leading to secondary caries [29,47].

Bioactive adhesives stabilize the hybrid layer through multiple mechanisms. They often contain MMP inhibitors, such as chlorhexidine, benzalkonium chloride, or zinc ions, which inactivate collagen-degrading enzymes and preserve hybrid layer integrity. Functional monomers like 10-MDP chemically bond with calcium ions in dentin, forming a hydrolysis-resistant layer [48,49,50,51]. Additionally, the continuous release of calcium, phosphate, and fluoride ions from bioactive fillers (e.g., bioactive glass, nano-hydroxyapatite) promotes remineralization of the hybrid layer over time. This ongoing mineral deposition reinforces the bond, enhances resistance to hydrolytic degradation, and helps prevent carious lesion progression. Together, these mechanisms preserve the hybrid layer, minimize adhesive failure and bacterial infiltration, and significantly extend restoration longevity.

## 4. Overview of Bioactive Dental Adhesives

### 4.1. Chemical Composition of Dental Adhesives

Direct composite restorations are one of the most frequently performed dental treatments. In this procedure, a dentist first applies an adhesive resin to the prepared tooth surface to ensure intimate contact and facilitate bonding. Adhesive resins, also known as bonding agents, are formulated to promote strong adhesion between the natural tooth structure (enamel or dentin) and the composite material [52].

Dental adhesives generally consist of four primary components: Resin monomers, initiators, additives, and solvents. Resin monomers such as Bis-GMA, HEMA, UDMA, and TEGDMA form the polymer network and dictate the adhesive’s mechanical strength, viscosity, and crosslinking density [53]. Initiators, including light-activated camphorquinone (CQ) and chemically activated benzoyl peroxide (BPO) with a tertiary amine, trigger polymerization, either on demand by light or autonomously, with dual-curing options available [54,55,56]. Additives enhance adhesive performance and durability; functional monomers like 10-MDP chemically bond to both resin networks and tooth hydroxyapatite [51,57], while bioactive fillers, such as nanoparticles of amorphous calcium phosphate, bioactive glass, or hydroxyapatite, promote ion release, remineralization, and potential antibacterial effects. Other additives, such as stabilizers (e.g., monoethyl ether hydroquinone and butylated hydroxytoluene), are included to prevent premature polymerization and to extend the shelf life of the adhesives [53]. Finally, solvents like ethanol, acetone, and water ensure the proper penetration of adhesive components during application [53]. The bioactive components are loaded either in the resin network or as additives.

### 4.2. Current Challenges in Dental Adhesive Systems

While the adhesive layer is essential, it is also considered the most susceptible area within the layered structure of a bonded restoration [2]. The poor durability of the adhesive can lead to pathway formation for bacterial infiltration and the formation of secondary caries after the composite resin restoration is placed in the patient’s mouth. To address the durability challenges, researchers have been developing more durable and hydrolytically stable adhesives by replacing hydrolysis-prone traditional monomers such as Bis-GMA and HEMA. In 2020, Dr. Sun and his team introduced a novel dental adhesive formulation using TEG-DVBE/UDMA, which demonstrated comparable bonding performance to conventional systems while offering significantly improved durability [6]. Beyond enhancing mechanical stability, incorporating therapeutic functionality into the adhesive layer would offer additional clinical benefits. The integration of bioactive materials into dental adhesives could enable therapeutic effects such as remineralization or antimicrobial action. These advancements have the potential to redefine restorative dentistry by improving both the longevity and biological performance of resin-based restorations.

### 4.3. Bioactive Agents in Dental Adhesive Systems

Many manufacturers have developed bioactive resin restorative materials that provide fluoride-releasing or antimicrobial effects to prevent the secondary caries problem. However, the first intimate layer to the cavity is not the composite resin restoration layer, but rather the adhesive layer. Hence, the development of bioactive dental adhesives is crucial for advancing the future of restorative dentistry. The modification of dental adhesive to achieve bioactivity could be introduced by two components. The first and most common modification is the additives. Many studies have been adding bioactive particles to the dental adhesive to achieve remineralization, antibacterial, and/or regeneration. The details of each bioactive particle will be discussed in the following section. Second, the modification of the monomer. Some studies switched out the monomer to gain the bioactivity [58,59].

Traditional dental adhesives primarily offer mechanical retention, lacking therapeutic interaction. They neither release remineralizing ions nor prevent microbial infiltration, making restorations susceptible to recurrent decay, especially at the adhesive-tooth interface [60]. Thus, the bioactive approach represents a more holistic and long-term strategy for achieving restorative success. Over the years, dental adhesives have evolved beyond micro-mechanical retention to enhance chemical bonding and simplify protocols from three-step bonding to one-step universal adhesive systems. Future development aims to integrate therapeutic functions, such as remineralization, antimicrobial, and regenerative effects, often by integrating bioactive components. This section will explore prominent bioactive compounds currently used in dental adhesives. Table 1 summarizes the current bioactive components and their respective functions in bioactive dental adhesives.

**Table 1:** Bioactive Materials Used in Dental Adhesives.

*Types*	*Materials*	*Bioactive* Function *	*Mechanism of* *Action*	*Limitations*	*Adhesive* *Integration*
*Section 4.3.1* * Calcium Phosphate Particles *	Amorphous Calcium Phosphate (ACP) [14]	Remineralization	Ion Exchange; Disruption of bacterial cell membrane	Limited long-term stability	Additives
	Hydroxyapatite Nanoparticles (n-HAp)	Antimicrobial Remineralization	Ion Exchange; Disruption of bacterial cell membrane	Prone to degradation upon moisture exposure	Additives
*Section 4.3.2* * Bioactive Glass (BAG) *	Calcium Sodium Phosphosilicate [61]	Remineralization Antimicrobial Regenerative	Ion exchange elevates pH and activates reparative cellular responses.	Potential to interfere with resin components	Additives
*Section 4.3.3* * Antimicrobial Agents *	Nanoparticles of silver (NAg) [62]	Antimicrobial	Disrupt bacterial membranes and generate ROS	May cause discoloration and cytotoxicity at high doses	Additives
	Quaternary ammonium dimethacrylate (QADM) [58]	Antimicrobial	Contact kills by disrupting bacterial membranes	Limited sustained antimicrobial effect.	Resin Monomer
	Methacryloyloxydodecylpyridinium bromide (MDPB) [59]	Antimicrobial	Sustained contact-based membrane disruption	Reduce the degree of conversion	Resin Monomer
	Nisin peptide [63]	Antimicrobial	Form pores in bacterial membranes	Unstable; enzyme-prone degradation	Additives
	Glutaraldehyde [64]	Antimicrobial	Cross-links bacterial proteins, leading to cell death	Tissue toxicity; polymerization interference	Additives
	Chlorhexidine [65]	Antimicrobial	Disrupts membranes; precipitates cytoplasm	Short-term effectiveness; Leaching over time	Additives

* Bioactive functions; Remineralization = Material can release ions that can deposit on the demineralized tooth structure. Antimicrobial = Material can kill cariogenic bacteria or inhibit their growth. Regenerative = Material can stimulate cells in the pulp.

#### 4.3.1. Calcium Phosphate Particles

Calcium and phosphate ions are crucial for **remineralization** processes. A variety of calcium phosphate-based materials have been explored for incorporation into dental adhesive systems, including amorphous calcium phosphate (ACP), calcium silicate, dicalcium phosphate dihydrate (DCPD), β-tricalcium phosphate (β-TCP), and nanosized-hydroxyapatite (n-HAp) [66]. These materials act as reservoirs of calcium and phosphate ions, which are gradually released into the surrounding environment under acidic conditions or cariogenic settings. Once the ions are released, they can precipitate into hydroxyapatite-like crystals on the dental hard tissue surfaces, thereby promoting remineralization. Moreover, calcium phosphate particles, especially calcium phosphate nanoparticles, can also occlude the dentinal tubule [67]. With this property, calcium phosphate was also utilized as a desensitizing agent [68].

Among bioactive compounds, amorphous calcium phosphate (ACP) is one of the most widely used and thoroughly studied. As a non-crystalline, metastable form of calcium phosphate, ACP is highly soluble and capable of rapid ion release. When incorporated into dental adhesives, it serves as an immediate source of calcium and phosphate ions, promoting mineral nucleation and crystal growth within demineralized dentin. Its high solubility offers the advantage of fast and substantial ion release, making it effective for remineralization. However, this same property poses a challenge: ACP tends to spontaneously convert into more stable crystalline phases, such as octacalcium phosphate or apatite, upon exposure to moisture [69]. This transformation can reduce its long-term bioactivity and compromise its clinical durability [68].

To overcome ACP’s instability, various stabilization strategies have been developed to preserve its amorphous state until ion release is functionally required. One approach involves the use of casein phosphopeptides (CPP), which bind to ACP and form nanoclusters (CPP-ACP) that stabilize the amorphous phase while enabling controlled ion release [70,71]. Another method uses citrate ions (Cit-ACP) to inhibit crystallization by chelating calcium ions, preventing premature aggregation into crystalline forms [72]. Both CPP-ACP and Cit-ACP have shown enhanced enamel and dentin remineralization in laboratory and clinical studies, making them promising candidates for incorporation into next-generation dental adhesive systems [67]. ACP is also available in nano-sized form. Recent advancements have demonstrated that nano-sized ACP particles exhibit significantly higher efficiency in promoting enamel and dentin remineralization compared to their larger-sized counterparts [73]. This enhanced efficacy is primarily attributed to the increased surface area of nanoparticles, which allows for better adhesion to demineralized surfaces and more effective ion delivery. Several in vitro studies have confirmed that nano-ACP formulations facilitate deeper mineral penetration and faster restoration of enamel microhardness than conventional ACP [73]. Therefore, the incorporation of nano-sized ACP in dental materials offers promising improvements in non-invasive treatment strategies for early carious lesions.

Nano-hydroxyapatite (n-HAp), a synthetic form of calcium phosphate, has also been investigated in dental adhesive systems due to its close resemblance to the mineral composition of natural enamel and dentin (Ca_10_(PO_4_)_6_(OH)_2_; Ca:P ratio of 1.67). These nanoparticles serve as a bioavailable source of calcium and phosphate ions, promoting remineralization at the adhesive interface [74]. The process involves nucleation and growth of hydroxyapatite crystals, which fill demineralized areas, restoring mineral density, mechanical strength, and resistance to acid attack. Additionally, n-HAp reduces dentin permeability and occludes dentinal tubules, offering the added clinical benefit of alleviating dentin hypersensitivity [75]. In a study by Sadat-Shojai et al., hydroxyapatite was synthesized in nanorod form and added to dental adhesives at concentrations of 0.2–0.5 wt%, resulting in enhanced mechanical properties. However, the study did not assess the bioactivity of the modified dental adhesives. Additionally, the long-term stability of n-HAp within the adhesive matrix remains uncertain, as its gradual ion release may affect the mechanical integrity of the adhesive over time [76]. In addition to their remineralization properties, calcium phosphate particles have demonstrated antimicrobial potential. Nanoparticles such as ACP and HAp exhibit bacteriostatic, non-genotoxic activity by disrupting bacterial membranes. ACP, with its higher surface reactivity, causes significant external membrane damage, while HAp is more readily internalized and may inhibit bacterial resistance by interfering with efflux pumps. These mechanisms disrupt key membrane components in both Gram-positive and Gram-negative bacteria, offering added antibacterial benefits to their bioactive profile [77]. In summary, calcium and phosphate compounds, especially ACP and n-HAp, hold significant potential in advancing bioactive dental adhesives. Their capacity to promote remineralization, exert antimicrobial effects, and integrate with tooth structure represents a transformative step toward more biologically interactive and therapeutic dental adhesive systems.

Although experimental studies have shown promising results with calcium phosphate (CaP) containing dental adhesives, there are few, if any, such bonding agents are commercially available in the United States. CaP is more commonly incorporated into composites, liners, or cements rather than adhesives [78]. This limited translation may reflect challenges in achieving long-term stability, as the effects of aging and the durability of CaP-containing adhesives remain insufficiently studied [79].

#### 4.3.2. Bioactive Glass

Bioactive glass (BAG) is a silica-based sodium calcium phosphosilicate known for its excellent bioactivity and compatibility with dental tissues. One of the most studied formulations, Bioglass^®^ 45S5, comprises approximately 45 wt% SiO_2_, 24.5 wt% CaO, 24.5 wt% Na_2_O, and 6.0 wt% P_2_O_5_. Upon exposure to physiological fluids, BAG initiates surface reactions that release calcium and phosphate ions, leading to the formation of a hydroxycarbonate apatite (HCA) layer. This mineral closely mimics natural apatite in enamel and dentin, facilitating effective remineralization of demineralized tooth structures [80]. When bioactive silicate glasses encounter body fluids, they undergo a sequence of chemical reactions that culminate in the formation of a hydroxycarbonate apatite (HCA) layer. This layer is chemically and structurally like the natural apatite phase found in bone. The remineralization process, originally described by Hench, occurs in five distinct stages [81].

1. **Ion Exchange:** The glass releases sodium and calcium ions into the surrounding fluid, while hydrogen ions (H^+^ or H_3_O^+^) enter the glass. This raises the local pH and begins breaking the silicon-oxygen (Si–O–Si) bonds.
2. **Silica Dissolution:** The breaking of Si–O–Si bonds releases silicon into the fluid as silanol (Si(OH)_4_) molecules.
3. **Silica Gel Layer Formation:** If the pH stays below 9.5, silanol molecules condense to form a porous silica gel layer on the glass surface, allowing further ion exchange.
4. **Calcium Phosphate Layer Formation:** Calcium and phosphate ions from both the glass and fluid accumulate on the silica gel, creating a layer of amorphous calcium phosphate (ACP).
5. **HCA Crystallization:** Carbonate ions incorporate into the ACP layer, which gradually crystallizes into hydroxycarbonate apatite (HCA), closely resembling the mineral phase of natural teeth and bone.

This process not only helps repair damaged tissue but also supports the integration of dental materials with natural tooth structure, making bioactive glass a powerful component in restorative dentistry. Beyond its remineralizing properties, BAG also plays an active role in **regeneration** by modulating cellular behavior. The ionic dissolution products from BAG can stimulate the expression of growth factor-related genes, enhance the differentiation of osteogenic cells, and support the deposition of bone matrix. In the context of dental tissue repair, these bioactive ions have been shown to induce the differentiation of odontoblast-like cells, thereby initiating reparative dentinogenesis, a critical process for healing and protecting pulp-dentin complexes following injury or decay [80,81].

In addition to its regenerative capabilities, BAG demonstrated **antimicrobial activity**, particularly against cariogenic pathogens such as *Streptococcus mutans*. According to a study by Xu et al., the release of ions from BAG resulted in a localized increase in pH, creating an environment that is inhospitable to acidogenic bacteria. This elevation in pH contributes to the suppression of bacterial biofilm formation and activity, thereby reducing the risk of secondary caries development around restorations [82]. Collectively, the multifunctional properties of BAG, encompassing remineralization, cellular stimulation, and antimicrobial action, make it an attractive and versatile component in advanced dental adhesive systems aimed at enhancing the longevity and therapeutic value of restorations.

With controlled use, BAG presents minimal biological risk. Studies have shown that BAG does not exhibit clinically significant cytotoxicity or genotoxicity [83]. A key limitation of incorporating BAG into adhesives is the potential interaction with other adhesive components, which must be assessed individually. Despite these challenges, BAG has been included in at least one commercially available adhesive, RE-GEN™ Universal Adhesive (Vista Apex, Racine, WI, USA).

#### 4.3.3. Antimicrobial Agents

Incorporating antimicrobial agents into dental adhesives targets the persistent challenges of biofilm formation at restoration margins. Various compounds have been investigated for this purpose. Silver nanoparticles (NAg) disrupt bacterial membranes and generate reactive oxygen species (ROS) to exert their effect. Quaternary ammonium compounds, such as quaternary ammonium dimethacrylate (QADM), kill bacteria on contact by damaging their membranes [58,62,84]. Among commercially available adhesives, Clearfil SE Protect Bond Primer (Kuraray Noritake Dental Inc., Tokyo, Japan) is one of the few that incorporate MDPB as a monomer resin. Nisin peptide, a naturally occurring antimicrobial, forms pores in bacterial membranes, especially targeting Gram-positive bacteria [63]. Glutaraldehyde acts by cross-linking bacterial proteins, causing cell death, while chlorhexidine disrupts membrane integrity and precipitates cytoplasmic contents. Beyond its antimicrobial role, chlorhexidine pre-treatment has also been reported to enhance bond strength and reduce degradation at the adhesive interface [64,65]. The integration of these agents into dental adhesive systems offers a promising strategy to inhibit bacterial colonization and reduce secondary caries risk [85].

#### 4.3.4. Multifunctionality

Some materials inherently offer multifunctionality, providing properties such as remineralization, antimicrobial effects, and tissue regeneration. Another strategy to achieve multifunctional dental adhesives involves combining single-function components, either materials with similar functions to boost efficacy or those with distinct therapeutic roles to broaden benefits. For example, Dr. Hockin H. K. Xu and colleagues studied the combination of quaternary ammonium dimethacrylate (QADM), an antimicrobial monomer, with silver nanoparticles (NAg), antimicrobial fillers [22]. This combination significantly enhanced antimicrobial efficacy without compromising adhesive mechanical properties. Building on this, they developed an experimental adhesive incorporating nanosized amorphous calcium phosphate (NACP) for remineralization alongside NAg for antimicrobial activity. This adhesive demonstrated extensive NACP infiltration into dentinal tubules and exhibited a substantially stronger inhibitory effect on cariogenic biofilms compared to the Scotchbond™ multi-purpose control. Notably, lactic acid production by biofilms in NAg-containing groups was reduced to one-quarter of that in the control, highlighting a potent suppression of cariogenic activity [86].

## 5. Trends in Bioactive Dental Adhesives Development

Recent innovations in dental adhesives have transformed them from simple bonding agents into bioactive materials capable of interacting with and protecting tooth structures. These advanced adhesives provide sustained protection by adapting to the dynamic conditions of the oral environment. However, traditional methacrylate-based resin networks, containing ester bonds in monomers such as Bis-GMA and HEMA, remain susceptible to hydrolytic and enzymatic degradation, leading to network breakdown and reduced longevity. As a result, hydrolysis-resistant formulations, including Bis-GMA-free and HEMA-free networks, have gained increasing attention for their superior durability. Such stable resin matrices are essential for the development of bioactive adhesives that combine long-term mechanical resilience with therapeutic functionality. These advanced resin systems in adhesives and resin composites were discussed in detail in previous reviews [87,88,89]. While most bioactive components described in this section were developed using methacrylate-based resins due to their commercial availability, these strategies can be adapted to alternative resin systems.

The advancements result from the integration of biomaterials science, bio-nanotechnology, and biomimetic engineering [90,91,92]. The incorporation of bioactive glass (e.g., 45S5), functionalized bioactive glass, and ion-releasing polymers enables the controlled release of calcium, phosphate, and fluoride ions, thereby enhancing enamel and dentin remineralization and helping to prevent secondary caries and their progression [93]. Nanotechnology has further improved these adhesives, with nano-hydroxyapatite improving mechanical strength, bonding performance, and exhibiting antimicrobial properties to inhibit biofilm formation [94,95,96,97,98,99]. Moreover, smart stimuli-responsive dental adhesives were also reported to demonstrate real-time protection by releasing ions in acidic/cariogenic environments, effectively countering tooth demineralization and promoting remineralization [28,100].

Over the past decade, significant development has been reported with the introduction of self-healing polymers, which have demonstrated the capability to repair microcracks, thereby extending the lifespan of adhesive bonds. The use of a widely recognized functional monomer has enhanced chemical bonding to hydroxyapatite, improving the strength and durability of the adhesive interface [57]. In addition, antimicrobial agents such as chlorhexidine and quaternary ammonium compounds were incorporated to inhibit enzymatic degradation, bacterial growth, and biofilm formation at the tooth and adhesive interface, thereby reducing the risk of bond failure [101,102]. At the same time, emerging eco-friendly strategies emphasized the use of natural antimicrobials, regenerative materials, and biocompatible formulations [103,104,105]. These developments aimed not only to restore tooth structure but also to maintain and promote long-term oral health.

Research on bioactive dental adhesives can be categorized into four general areas, including **nanotechnology-based adhesives** for improved strength and antimicrobial action; **smart polymers and hydrogel-based adhesives** that respond to oral conditions; **enzymatic inhibitors** to prevent collagen degradation and extend longevity of the bond; and **clinical performance studies** assessing their durability and biocompatibility in clinical settings. The first three categories reflect the evolving functional potential of these materials. Table 2 provides an integrated overview of key components, nanomaterials, smart polymers, hydrogels, and enzymatic inhibitors, highlighting their primary roles and benefits in improving adhesive performance and therapeutic value. Table 3 summarizes the key clinical performance attributes, offering a clear overview of current directions in clinical application.

### 5.1. Nanotech-Enhanced Dental Adhesive Systems

Nanotechnology has become a transformative strategy in advancing the properties of dental adhesives by enhancing their mechanical strength, biological performance, biocompatibility, and therapeutic functions. Nanoparticles, typically measuring between 1 and 100 nanometers, exhibit a high surface-area-to-volume ratio, allowing for more effective interactions with both the tooth surface, adhesive, and resin matrix interface [106,107,108]. These enhanced functionalities promote improved resin infiltration into collagen networks and influence the formation of a strong, durable, and more stable hybrid layer, particularly in demineralized dentin [47,109]. Common nanofillers such as nano-hydroxyapatite (n-HAp) [76,94,110,111,112], nano-silica [113,114,115,116], nano-zirconia [117,118,119], and nano titanium dioxide [117,120] showed a critical role in enhancing the performance of dental adhesives. They contributed to improving mechanical properties, including bond strength, elastic modulus, and greater fracture and wear resistance. Their nanoscale size allows for better dispersion within the adhesive matrix and tooth surface, leading to stronger and more durable adhesive interfaces. Especially, nano-hydroxyapatite demonstrated a key role in biomimetic remineralization by releasing calcium and phosphate ions, which promoted the restoration of mineral content in early carious lesions and improved dentin sealing, thereby enhancing the overall integrity and longevity of the adhesive interface [121,122].

Current research in dental adhesive technology has gradually more focused on incorporating antimicrobial nanoparticles, for example, silver [123,124], zinc oxide [23,46], copper oxide [125,126,127], and chitosan [128,129], which disrupt bacterial cell membranes and inhibit biofilm formation at the tooth-adhesive interface. These antimicrobial properties are decisive for preventing secondary caries progression and enhancing the longevity of the restorations. In addition, the development of pH-responsive nanomaterials is gaining momentum. These materials have been designed to release therapeutic ions in acidic/cariogenic environments, allowing adhesives to neutralize acidic conditions and prevent demineralization and thus, support real-time remineralization.

Advanced nanostructures, including dendrimers [130,131], nanogels [132,133,134], and nanotubes [135,136,137], were also under investigation for their potential in controlled drug delivery, self-healing mechanisms, and improved interaction with dental tissues. While both in vitro and in vivo studies have demonstrated promising results, several challenges remain, particularly in ensuring long-term biocompatibility, minimizing cytotoxic effects, and satisfying regulatory requirements for clinical use. Continued interdisciplinary collaboration is essential to overcome these barriers and successfully integrate these nanotechnologies into standard dental practice.

### 5.2. Smart Polymers and Hydrogels-Based Therapeutic Dental Adhesives

Smart polymers and hydrogels have emerged as next-generation materials in dental adhesive systems, owing to their ability to respond dynamically to environmental stimuli in the oral cavity. Unlike conventional adhesives, these advanced systems enable site-specific and time-dependent therapeutic functions, such as pH buffering, ion release, drug delivery, and self-repair capacity [138,139,140]. Stimuli-responsive polymers release remineralizing agents like calcium (Ca^2+^), phosphate (PO_4_^3−^), and fluoride (F^−^) in response to acidic conditions caused by bacterial activity, promoting enamel repair and caries resistance [25,28,100]. Self-healing polymers based on vitrimer chemistry enable dynamic bond exchange reactions, allowing the adhesive layer to reorganize and repair microcracks generated by mechanical or thermal stress. This maintains interfacial integrity and extending the longevity of restorations [141,142,143].

Hydrogels, composed of hydrophilic polymer networks, offer excellent biocompatibility and the ability to mimic the extracellular matrix, making them ideal for interactions with the dentin–pulp complex [144]. They can also act as drug delivery reservoirs, providing sustained release of antimicrobials (e.g., chlorhexidine [145], silver nanoparticles [98]), enzymes [146], or growth factors [147]. Thermo-responsive hydrogels, such as PNIPAAm, undergo physical state transitions at body temperature, enhancing adhesive adaptability and curing efficiency [148,149].

Functionalized hydrogels like GelMA (gelatin methacryloyl) and PNIPAAm were being explored for dual-curing capabilities and temperature responsiveness [150,151]. Chitosan-based hydrogels further contribute antimicrobial activity and mucoadhesive properties, improving tissue contact and reducing bacterial colonization while enabling controlled drug release [43]. Injectable and enzyme-responsive hydrogels enable precision in minimally invasive procedures and allow targeted therapeutic release triggered by specific enzymatic activity at infection sites [11,152,153,154]. Moreover, amorphous calcium phosphate (ACP)/Nano-HA hydrogels facilitate calcium and phosphate ion delivery at the adhesive interface, enhancing remineralization and reducing dentin hypersensitivity, key factors that contribute to long-term restoration success [19,155].

### 5.3. Enzymatic Inhibitors Incorporated Bioactive Dental Adhesives

The incorporation of enzymatic inhibitors into bioactive dental adhesives has represented a critical advancement aimed at enhancing the durability and integrity of resin-dentin bonds. Enzymatic degradation, particularly by host-derived matrix metalloproteinases (MMPs), significantly contributes to the breakdown of the hybrid layer over time, leading to adhesive failure and restoration loss. These enzymes are activated during acid-etching or through bacterial activity and gradually degrade the exposed collagen matrix. To mitigate this, dental adhesives have been formulated with enzyme-inhibiting agents that stabilize the hybrid layer and protect the collagen framework from proteolytic attack [29,156,157].

Matrix metalloproteinase inhibitors, such as chlorhexidine [48], galardin [158], and benzalkonium chloride [49,159], are among the most extensively studied compounds for this purpose. These inhibitors can be incorporated directly into primers or adhesives, where they act by chelating the zinc ions required for MMP activity or by modifying enzyme conformation. Cysteine cathepsin inhibitors, including epigallocatechin gallate (EGCG) and tannic acid, offer complementary protection by targeting additional enzymatic pathways involved in dentin degradation [160,161]. Notably, the use of synthetic peptides and enzyme-inhibiting polymers is also being explored to provide more targeted, controlled, and long-lasting inhibition within the adhesive interface [152].

**Table 2:** Advanced Components in Bioactive Dental Adhesives: Nanomaterials, Smart Polymers, Hydrogels, and Enzymatic Inhibitors.

*Types*	*Agents*	*Target Functions*	*Added Benefits*
* Nanomaterials *	Nano-Hydroxyapatite (n-HAp) [76,94,110,111,112]	Biomimetic remineralization	Facilitates mineral deposition and reduces hypersensitivity
	Nano-Silica (SiO_2_) [113,114,115,116]	Mechanical reinforcement	Boosts bond strength, wear resistance, and marginal seal
	Nano-Zirconia (ZrO_2_) [117,118,119]	Toughening agent	Enhances fracture toughness and mechanical stability
	Titanium Dioxide (TiO_2_) [117,120]	Mechanical reinforcement	Increases bond strength; reduces microleakage and microbial adhesion
	Silver Nanoparticles (NAg) [123,124]	Antimicrobial	Suppresses biofilm; prevents recurrent caries
	Zinc Oxide Nanoparticles [23,46]	Antimicrobial and anti-inflammatory	Inhibits microbial colonization; modulates inflammation
	Copper Oxide Nanoparticles [125,126,127]	Antimicrobial	Boosts antimicrobial activity and bond durability
	Chitosan Nanoparticles [128,129]	Antimicrobial; drug delivery carrier	Facilitates healing with controlled drug release
	Dendrimers [130,131]	Self-healing	Enables pH-driven ion release and self-repair of microcracks
	Nanogels [132,133,134]	Smart delivery system	Enables pH-responsive ion and drug release
	Nanotubes (e.g., Halloysite) [135,136,137]	Therapeutic agent reservoirs	Enables prolonged antimicrobial release
*Smart Polymers* */Hydrogels*	pH-Responsive Polymers [25,100]	Ion release in acidic pH	Promotes remineralization and acid protection
	Diels–Alder/Self-Healing Polymers [142,143]	Autonomous microcrack repair	Improves longevity and strength at the adhesive interface
	Enzyme-Responsive Systems [146]	Targeted therapeutic release	Release agents in response to bacterial enzymatic action
	Gelatin Methacryloyl (GelMA) [150]	Light-curable hydrogel matrix	Enables moisture-resistant curing and tissue bonding
	Thermoresponsive Hydrogels (PNIPAAm) [151]	Thermoresponsive viscosity	Facilitates handling and better intraoral adaptation
	Chitosan-Based Hydrogels [43]	Antimicrobial, drug delivery carrier	Inhibits microbe; facilitates prolonged therapeutic delivery.
	ACP/nano-HAp Hydrogels [19,155]	Calcium/phosphate delivery	Enhances remineralization; decreases hypersensitivity
* Enzymatic * * Inhibitors *	MMP Inhibitors (e.g., Chlorhexidine, Galardin) [49,158,159]	Inhibits matrix metalloproteinases	Preserves hybrid layer; reduces adhesive degradation
	Cathepsin Inhibitors (e.g., EGCG, Tannic Acid) [160,161]	Blocks collagen-degrading cathepsins	Strengthens bond over time; protects interface integrity
	Peptide-Based Inhibitors (e.g., Synthetic MMP-inhibitory peptides) [152]	Selective MMP inhibition	Provides sustained enzyme inhibition with low toxicity
	Bioactive Fillers + Inhibitors (e.g., n-HAp + ACP) [162,163].	Dual action: remineralization and enzymatic inhibition	Reinforces framework and inhibits enzymatic degradation

Moreover, these enzymatic inhibitors have often been synergistically combined with bioactive fillers like nano-hydroxyapatite or calcium phosphate to provide dual functionality, enzymatic protection, and remineralization [162,163]. This combination promotes both mechanical stability and biological healing of the tooth structure. While these innovations offer promising benefits, such as reduced marginal leakage, enhanced bond longevity, and decreased postoperative sensitivity, several challenges remain. These include ensuring sustained inhibitor activity, preventing interference with polymerization, and maintaining biocompatibility. Ongoing research focuses on optimizing delivery methods, such as microencapsulation and polymer-bound inhibitors, to achieve sustained and controlled release without compromising the adhesive’s mechanical properties.

### 5.4. Clinical Performance Studies

Clinical performance studies are essential for validating the real-world efficacy, durability, and therapeutic impact of bioactive dental adhesives. Unlike in vitro evaluations, these studies assessed how adhesives perform in the complex oral environment, accounting for variables such as saliva, pH fluctuations, patient habits, and operator techniques. The key metrics investigated include bond strength longevity, caries prevention, antimicrobial efficacy, and patient-reported outcomes such as postoperative sensitivity and esthetic satisfaction.

Long-term bond strength was a primary focus, with studies indicating that bioactive dental adhesives can maintain adhesion to enamel and dentin over several years due to ongoing ion release and hybrid layer stabilization [164]. Materials incorporating bioactive glass (BAG) and nano-hydroxyapatite (nHAp) have demonstrated promising results in promoting remineralization, sealing dentinal tubules, and reducing marginal microleakage [165,166,167,168]. These benefits have been translated into better marginal integrity, helping to prevent secondary caries and extend the functional life of restorations. Clinical trials also showed that the sustained release of fluoride, calcium, and phosphate ions contributed to a reduction in postoperative sensitivity and enhanced biocompatibility [169,170,171,172]. Additionally, adhesives with embedded antimicrobial agents like zinc oxide or silver nanoparticles showed reduced bacterial colonization at the restoration interface [173,174].

However, variability in outcomes due to patient-specific factors (e.g., oral hygiene, cavity location, moisture control) remains a challenge. Comparative studies continued to examine how bioactive dental adhesives stack up against traditional systems, with most reported superior or equivalent performance in high-risk environments. These findings supported their growing adoption in clinical practice, though further long-term, multicenter trials are necessary to fully establish their efficacy and optimize application protocols.

Dental adhesives have evolved from passive bonding agents to multifunctional materials that actively enhance restoration longevity, resist degradation, and promote maintenance of tooth structure. Advances in nanotechnology, smart polymers, enzymatic inhibitors, and clinical validation have collectively improved bond strength, antimicrobial action, and remineralization capacity. However, future research should focus on enhancing the durability and responsiveness of these systems under dynamic oral conditions. Developing multifunctional adhesives with precise, stimuli-responsive release of therapeutic agents will further improve clinical outcomes. Integration of regenerative technologies like peptides or stem cell-compatible matrices holds great promise. Standardized in vitro and long-term clinical studies are essential to ensure safety, efficacy, and practical adoption in clinical settings.

**Table 3:** Evaluation of Bioactive Dental Adhesives: Clinical Functionality and Performance Parameters.

*Engineered Performance*	*Experimental Outcomes*	*Clinical Benefits*
*Remineralization* [165]	BAG and nHAp induce apatite formation, restoring mineral content in dentin/enamel	Aids tooth preservation and repair
*Postoperative Sensitivity* [169]	Ion release mitigates dentinal hypersensitivity in deep cavities	Improves comfort and compliance.
*Secondary Caries Prevention* [169]	Fluoride/calcium ions prevent demineralization and bacterial ingress	Strengthens caries defense at margins
*Biocompatibility* [172]	Safe and well-tolerated; fluoride and BAG offer low-toxicity therapeutic effects	Enables safe application in pulp and deep cavities
*Bond Strength* [175]	Strong initial bond; ion release boosts durability in moist conditions	Ensures reliable adhesion in challenging clinical settings
*Marginal Integrity* [176]	Minimizes microleakage with enhanced sealing and adaptation	Prevents bacterial ingress and recurrent caries
*Longevity* [177]	Stable adhesion for 5+ years, dependent on hygiene and site	Minimizes long-term replacement needs

## 6. Current Challenges and Limitations in Advanced Bioactive Dental Adhesive Systems

Compatibility of bioactive additives with resin networks is a major challenge in the development and clinical application of bioactive dental adhesives These fillers often differ significantly from the resin in surface chemistry, polarity, and reactivity, which can interfere with polymerization, disrupt network formation, and compromise adhesive performance [178,179]. Incompatibility can lead to nanoparticle agglomeration, poor dispersion, or phase separation, resulting in heterogeneous structures with weak interfacial bonding. This undermines critical properties such as mechanical strength, dimensional stability, and water resistance. Additionally, some bioactive components may react with initiators or free radicals during light curing, reducing the degree of conversion and yielding unstable polymer networks [180,181,182]. To address these issues, surface functionalization techniques such as silanization and methacrylate grafting are used to improve filler–resin interaction. Resin formulation strategies, including the use of compatible co-monomers and optimized monomer-to-filler ratios, further enhance dispersion and interfacial adhesion without compromising bioactivity [183,184]. Therefore, it is essential to achieve uniform distribution and stable integration of bioactive agents to maintain mechanical integrity and ensure sustainable ion release, antimicrobial action, and remineralization. Furthermore, enhancing the intermolecular chemistry between fillers and resins remains a key priority for developing sustainable and medically effective bioactive dental adhesives.

Enhancing the bioactivity of dental adhesives often involves incorporating inorganic fillers or hydrophilic agents that promote ion release and remineralization. However, these additives can compromise mechanical properties such as tensile strength, flexural modulus, fracture toughness, and wear resistance by increasing water sorption and introducing matrix heterogeneity, which weakens structural integrity and durability [132,178]. Striking an optimal balance between therapeutic functionality and mechanical performance remains a key challenge in materials science. While bioactive components enhance antimicrobial effects and support tissue remineralization, they may also reduce bond strength and resistance to mechanical stresses. Current research focuses on optimizing filler size, concentration, and surface modification techniques to preserve adhesive durability while retaining bioactive benefits.

The long-term success of bioactive dental adhesives depends on their ability to resist hydrolytic and enzymatic degradation while maintaining strong, stable bonds. While ion-releasing dental adhesives show promise in dentin remineralization and hybrid layer preservation, their durability under the complex conditions of the oral environment remains a major concern. Factors such as thermal cycling, enzymatic activity (particularly from matrix metalloproteinases and cathepsins), and repeated mechanical loading contribute to collagen matrix degradation within the hybrid layer. This leads to reduced bond strength, increased microleakage, and ultimately, restoration failure [185,186,187]. Therefore, the development of dental adhesives that can sustain both mechanical and biological functions while resisting hydrolysis and enzymatic breakdown is essential for improving long-term restoration performance and clinical reliability.

Another key limitation of many bioactive dental adhesives is their compatibility with established clinical protocols and the lack of randomized controlled trials to substantiate their clinical efficacy. These formulations often require modifications to standard procedures such as etching, priming, or curing, which can complicate chairside application and increase the learning curve for practitioners. For instance, some may demand longer curing times, specific handling techniques, or alternative solvent systems to maintain bioactivity without compromising bond strength. Such deviations from conventional workflows can hinder clinical efficiency and limit widespread adoption [9,57]. Therefore, developing user-friendly bioactive dental adhesives that integrate seamlessly into existing protocols without requiring significant procedural changes is essential for ensuring clinician acceptance and translating laboratory advances into routine dental practice.

## 7. Conclusions and Future Perspective

Advances in dental adhesive technology are driving the development of multifunctional systems that not only achieve strong and durable adhesion but also deliver therapeutic benefits such as remineralization and antimicrobial protection. Key strategies include incorporating functional bioactive monomers like 10-MDP for chemical bonding to tooth calcium, ion-releasing fillers for sustained mineral delivery, and enzyme inhibitors to preserve the collagen network within the hybrid layer. Emerging approaches, such as stimuli-responsive and self-healing materials, offer the potential to adapt to the oral environment and repair microdamage, further enhancing longevity. Future work should optimize formulations for controlled ion release and validate their performance through long-term in vivo trials, especially in high-caries-risk populations. To address the current lack of clinical data, well-designed randomized controlled trials (RCTs) and longitudinal cohort studies are needed, with clear endpoints including restoration survival, secondary caries incidence, and biological effects at the adhesive–tooth interface. Standardized methodologies and consistent outcome measures will be crucial for generating comparable, high-quality evidence. Bio-mimetic designs that mimic natural remineralization and form a sealed, acid-neutralizing, antimicrobial interface hold promise for preventing bacterial colonization, reducing microleakage, and mitigating secondary caries. Collectively, these innovations aim to extend restoration lifespan and improve long-term oral health outcomes, providing a clear roadmap for translating bioactive adhesive research into clinical practice.

## Data Availability

No new data were created or analyzed in this study. Data sharing does not apply to this article.
